# Development and evaluation of a recreational water quality index for the Red Sea Coastline, Saudi Arabia

**DOI:** 10.1038/s41598-026-54623-9

**Published:** 2026-05-24

**Authors:** Talal Almeelbi, Fawaz Alghamdi

**Affiliations:** 1 Department of Environment, Faculty of Environmental Sciences, King Abdulaziz University, Jeddah, 21589 Saudi Arabia; 2 National Center for Environmental Compliance, Riyadh, Kingdom of Saudi Arabia

**Keywords:** Coastal pollution, Microbiological indicators, Recreational water quality index (RWQI), Red sea, Water quality assessment, Environmental sciences, Microbiology, Water resources

## Abstract

Recreational water quality in coastal areas is vital for public health, especially in urban regions. In this study, a recreational water quality index (RWQI) tailored to the Red Sea coastline in Jeddah, Saudi Arabia, was developed to assess water quality at popular beaches. The samples were collected from three different locations. To develop the RWQI, relevant water quality parameters, such as the pH, dissolved oxygen (DO), total nitrogen (TN), total phosphorus (TP), *Escherichia coli* (*E. coli*), and enterococci, were identified and weighted on the basis of their significance for recreational water safety. These parameters were rated according to the World Health Organization (WHO) and Saudi Arabia Ambient Water Quality Standards (SAAWQS). The results were then aggregated into a single index score reflecting the overall water quality. The results revealed that Obhur Al-Shamaliya exhibited generally good water quality, particularly in winter, with index scores ranging from 83 to 98, meeting both the WHO and Saudi standards. All three sites recorded poorer water quality during the second sampling period in summer, with nutrient enrichment and *E. coli* counts exceeding permissible limits (2,300, 3,875, and 2,58.13 CFU/100 mL, respectively), resulting in an index classification as unsuitable for recreational purposes. The urban runoff sites were at high risk, with elevated pollution. Moreover, a one-way ANOVA test confirmed that the seasonal variation in the RWQI was statistically significant (F = 8.29, *p* = 0.045), emphasizing the influence of environmental factors, such as temperature and runoff, on recreational water quality. These findings underscore the need for season-specific monitoring and management strategies in coastal recreational areas.

**Supplementary Information:**

The online version contains supplementary material available at 10.1038/s41598-026-54623-9.

## Introduction

Water quality, especially in recreational areas, has attracted significant attention in recent years because of the increasing number of water-related health issues and environmental challenges^1–5^. Coastal regions, such as those along the Red Sea in Saudi Arabia, are popular destinations for recreational activities, including swimming, boating, and diving. However, the growing urbanization and industrial activities near these coastal areas have raised concerns about water pollution, particularly concerning microbial contamination, nutrient levels, and chemical pollutants^6–9^.

The correlation between the occurrence of gastrointestinal (GI) ailments among swimmers and fecal contamination indicators, such as enterococci and *E. coli*, is highlighted by the epidemiological history of recreational water use. These markers are typically employed; however, their ability to predict the presence of waterborne pathogens and the corresponding health effects is limited. The connection between these indicators and dangers to human health is complicated by several factors, such as the type of disease, environmental factors, and fecal indicator bacteria (FIB) behavior^10–12^.

The need for a reliable, localized assessment tool has become critical for maintaining safe water quality in recreational areas. One of the most effective methods for assessing water suitability for intended purposes is the water quality index (WQI), which integrates multiple water quality parameters into a single, easily interpretable score^13^. In 1960, Horton began classifying water quality with an emphasis on developing indices to gauge the effectiveness of pollution management initiatives^14^. Various researchers have attempted to develop WQIs based on different aggregation functions, each offering a distinct method to compute an overall water quality score from individual water parameters. Five main types of aggregation functions have been widely used: (a) arithmetic aggregation functions, (b) multiplicative aggregation functions, (c) geometric means, (d) harmonic means, and (e) minimum operators. Each function has its strengths and weaknesses, depending on the application and characteristics of the water body being assessed^15^.

The arithmetic aggregation function is among the most commonly used methods for calculating WQIs because of its simplicity and ease of interpretation. The core idea of this function is to sum the weighted sub-index values of each parameter, assuming that each parameter contributes independently to the overall water quality. The multiplicative aggregation function offers a different approach by multiplying the sub-indices of each parameter rather than summing them. This method places greater emphasis on low values, as a low score for one parameter will have a disproportionate effect on the overall WQI, thus highlighting the importance of addressing poor-quality parameters^16^. On the other hand, the geometric mean offers a compromise between the arithmetic and multiplicative aggregation functions. It is particularly useful when the goal is to balance the influence of high and low values across multiple parameters, reducing the distortion caused by extreme values^17^. The harmonic mean is designed to emphasize the lowest values in a dataset, making it highly sensitive to poor water quality for any one parameter. This method is particularly useful when it is crucial that none of the parameters fall below a certain minimum threshold, such as in drinking water or habitats for sensitive species^18^. The minimum operator is an aggregation function that selects the lowest sub-index value among all the parameters, making it the overall WQI. This method is based on the idea that the worst aspect of water quality should define the overall status, ensuring that any significant issue is immediately highlighted^19^.

WQIs are highly effective at simplifying complex water quality data into a format that is easy to interpret. This makes them invaluable for communicating the status of water quality to nonspecialists, raising public awareness, and informing policy decisions. By consolidating large datasets into a single value, WQIs make it simpler to track trends over time and to compare the quality of different water bodies^20^. Moreover, WQIs can be customized to meet specific water quality management objectives, whether for drinking water, recreation, or ecosystem health. This adaptability allows indices to be developed that reflect the distinct conditions and regulatory standards of various regions and water uses^16^. Additionally, WQIs assist in identifying pollution sources and assessing the success of water management practices^21^.

Given the unique environmental conditions and pollution sources affecting the Red Sea coastline, a new or adapted RWQI is clearly needed. While existing WQIs are valuable, they may not fully address the specific challenges posed by the local environment, such as high levels of discharged untreated sewage and industrial pollutants^6,22,23^. A localized RWQI for Jeddah would need to account for the specific pollutants prevalent in the region, including nutrient loading and microbial contaminants. Additionally, the recreational activities commonly undertaken in these waters should be considered, ensuring that the index accurately reflects the potential health risks associated with these activities^24,25^.

The aim of this study is to develop a comprehensive recreational water quality index tailored to the unique environmental conditions of the red sea coastline in Jeddah, Saudi Arabia, and to apply it to evaluate the suitability of selected recreational sites. This study further aims to identify potential sources of pollution that influence coastal water quality and to provide recommendations for improved monitoring and management practices that ensure safe recreational use and protection of public and marine ecosystems.

## Materials and methods

### Development of the RWQI

The WQI is calculated using a weighted arithmetic mean method, providing a composite score that reflects the overall condition of the water. The index was calculated using the following mathematical approach:

- Selection of Water Quality Parameters: The first step involves selecting significant water quality parameters that represent the water condition.
- Development of a Rating Scale (Rs): A rating scale is developed for the range of values of each parameter. This scale, which varies from 0 to 100, categorizes water quality into different classes, from excellent to unsuitable for recreational purposes, on the basis of the concentration of each parameter.
- Estimating the Unit Weight of Each Indicator Parameter (Wi): Each parameter’s relative importance or weight is determined on the basis of its impact on water quality using the Analytic Hierarchy Process (AHP).
- Determining the Sub-index Value (Wi x Rs): For each water quality parameter, a sub-index value is calculated by multiplying its unit weight (Wi) by its rating (Rs) obtained from the developed scale.
- Aggregating the Sub-indices to Obtain the Overall WQI: The overall WQI is calculated by summing the products of the rating and unit weight of all the parameters. This sum provides a single value representing the water body’s overall quality^17^.

The mathematical formula for calculating the WQI is as follows:

$$\:WQI={\sum\:}_{i=1}^{n}(Wi\times\:R{s}_{i})$$

where:

Wi = unit weight of the ith parameter,

Rsi = rating of the ith parameter on the basis of its concentration,

n = number of parameters considered.

### Study area

This study focuses on three coastal areas designated for recreational activities in Jeddah, a major city situated on the Red Sea coastline in western Saudi Arabia. The selection of Jeddah as the study area offers several advantages. Jeddah’s coastal waters are extensively used for recreational activities, such as swimming, snorkeling, and fishing. Consequently, understanding water quality and identifying potential pollution sources are essential for safeguarding the health and safety of both residents and tourists engaged in these activities. Jeddah’s coastal environment is subject to various stressors, including urban development, industrial activities, wastewater disposal, and population growth^26–30^.

#### Data collection

Understanding the seasonal dynamics and environmental conditions of recreational water bodies is crucial for accurately assessing water quality. This study carefully examines both summer and winter conditions to capture the potential impact of seasonal variations on water quality in Jeddah’s coastal recreational areas. Compared with the winter months, the summer months, characterized by higher temperatures and increased recreational activities, often present a different set of challenges, which may result in reduced human activity but are subject to different environmental impacts. Seasonal variations have been shown to significantly influence water quality, as highlighted in studies that document shifts in microbial contamination, nutrient loading, and other pollutants depending on seasonal conditions^31^.

In this study, water samples were collected during both summer and winter to capture potential seasonal variations in water quality. For each season, three rounds of sampling were conducted at all the sites. The summer sampling rounds were carried out on 12.08.2023, 26.08.2023, and 16.09.2023, denoted as S1, S2, and S3, respectively. Similarly, the winter sampling rounds were conducted on 24.01.2024, 11.02.2024, and 09.03.2024, denoted as W1, W2, and W3, respectively. This approach allowed for comprehensive monitoring of both seasonal and site-specific fluctuations in water quality.

#### Sampling locations

**Fig. 1:**
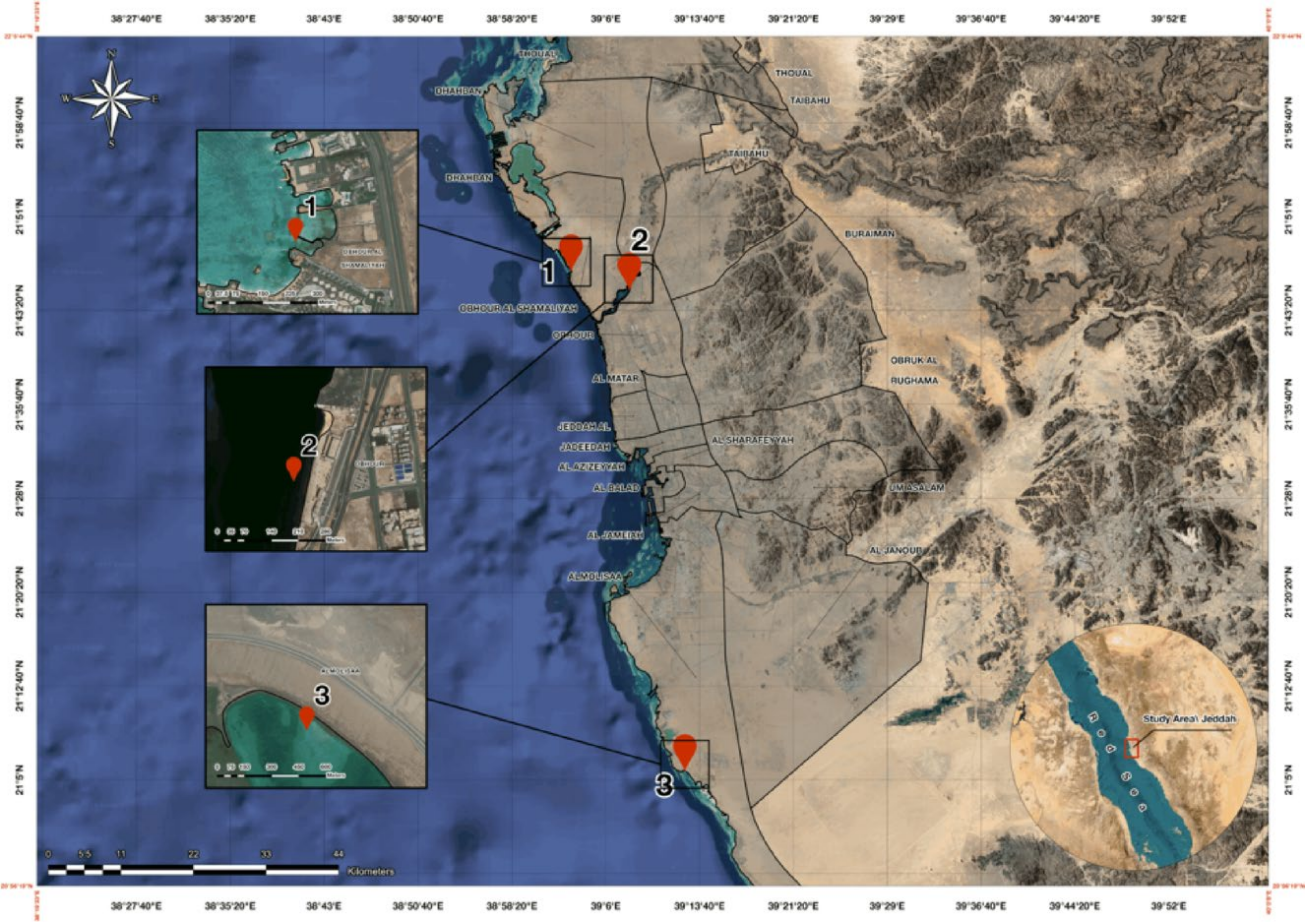
Locations of water sampling along the Jeddah coastline. The map was created by the authors using ArcGIS Pro version 3.5 (https://www.esri.com).

Water samples were collected from three key locations along the Jeddah coastline: Obhur Al-Shamaliya (L1), Obhur Al-Janoubiyah (L2), and Al-Saif Beach (L3) in southern Jeddah, as shown in Fig. 1.

### Analytical methods and quality control

Water quality parameters were analyzed following standard procedures to ensure the accuracy and comparability of the results. The pH level was measured according to EPA Method 150.1 using a portable multimeter (HACH). Dissolved oxygen was measured using EPA Method 360.1. Turbidity was measured using EPA Method 180.1. Total phosphorus (TP) and total nitrogen (TN) were measured according to DIN 38,405 D11-4 and Hach LCK-338, respectively, using a HACH DR5000 spectrophotometer. The method detection limits (MDLs) were 0.05 mg/L for TN and 0.01 mg/L for TP. Total ammonia was measured using EPA Method 350.1. Microbiological indicators included total coliform and *Escherichia coli* (*E. coli*) concentrations measured using APHA 9215 D, and enterococci levels were determined using EPA Method 1600^32,33^. The detection limit was 1 CFU/100 mL for both *E. coli* and enterococci.

Water samples were collected at a depth of 0.3–0.5 m below the surface. For nutrient analysis, grab samples were collected in acid-washed HIDPE bottles (250–500 ml), preserved with concentrated H_2_SO_4_ to a pH < 2, and stored at < 6 °C. The microbiological samples were obtained in sterile 120 ml bottles containing sodium thiosulfate, transported on ice and analyzed within 6–8 h.

To ensure the accuracy and reliability of the data, a series of rigorous quality control (QC) and quality assurance (QA) protocols were applied:

- Calibration of Instruments: All instruments used, including multimeters and spectrophotometers, were calibrated before each sampling session according to the manufacturer’s specifications to ensure precise measurements.
- Control and Blank Samples: Control samples—with known concentrations—and blank samples (deionized water) were analyzed alongside the water samples to detect any contamination or procedural errors, ensuring the integrity of the results.
- Replicates and Consistency Checks: Multiple replicates of each sample were analyzed to ensure consistency, allowing for the identification of outliers and the validation of data accuracy.
- Standard Operating Procedures (SOPs): All analyses followed strict SOPs to minimize human error and ensure uniformity in the methods across all sample sites.
- An acceptable relative standard deviation (RSD) of ≤ 20% was used for duplicate measurements.

### Statistical analysis

To evaluate the seasonal variations in the RWQI values, one-way analysis of variance (ANOVA) was employed. Before the data were analyzed, they were assessed for adherence to essential statistical assumptions. The normality of RWQI distributions within seasonal groups (summer vs. winter) was confirmed using the Shapiro–Wilk test, whereas the homogeneity of variances across groups was verified using Levene’s test. Both assumptions were met, thereby justifying the application of the ANOVA to this dataset. As the comparison involved only two groups, post hoc analysis was deemed unnecessary. This methodological approach ensured the robustness and reproducibility of the statistical analyses.

## Results

### Development of the RWQI

#### Parameter selection

The key water quality parameters were prioritized in this study because of their significance in maintaining recreational water safety. These include microbial indicators (*E. coli* and enterococci), which are essential for assessing health risks related to fecal contamination. In addition, the following physicochemical parameters were measured: pH, dissolved oxygen, nutrient levels (total phosphorus and total nitrogen), turbidity, and total ammonia.

#### Rating scale (Rs)

The rating (Rs) for each parameter is determined on the basis of the observed values relative to the ranges provided in the rating scale (Table 1). This rating scale, developed following the methodology outlined in the previous section, is based on combined guidelines from the WHO and the SAAWQS. This collaboration ensures that the rating scale is finely tuned to both global health safety protocols and local environmental conditions, providing an accurate and regionally applicable measure of water quality.

For pH, a range between 6.5 and 8.5 is considered excellent, reflecting optimal conditions for recreational water. Dissolved oxygen concentrations above 5 mg/L are considered excellent, indicating a healthy aquatic environment. Turbidity values of 3 NTU or less are considered ideal. The scale specifies ranges for TN, *E. coli*, and other parameters, with each parameter’s range meticulously defined to reflect water quality from excellent to unsuitable for recreational purposes, as shown in Table 1.

For example, water with pH values less than 4 or greater than 11 or DO concentrations less than 3 mg/L falls into the category of unsuitable for recreational purposes, highlighting the stringent criteria set for ensuring water safety. Similarly, elevated levels of *E. coli* above 500 CFU/100 mL indicate severe contamination, rendering the water unsuitable for recreational use. Notably, concentrations classified as unsuitable for recreational purposes have a negative impact on public health.

**Table 1:** Rating scales (Rs) of the selected parameters.

Parameter	Excellent	Good	Poor	Unsuitable for recreational purposes
pH	6.5–8.5	> 8.5 to ≤ 9 or < 6.5 to ≥ 5	< 5 to ≥ 4 or > 9 to ≤ 11	< 4 or > 11
Dissolved oxygen (DO)	≥ 5	≥ 4.0 to < 5.0	≥ 3.0 to < 4.0	< 3
Turbidity (NTU)	≤ 3	> 3.0 to ≤ 10.0	> 10.0 to ≤ 50.0	> 50.0
Total phosphorus (TP mg/L)	≤ 0.02	0.02–0.03	0.03–0.05	> 0.05
Total nitrogen (TN mg/L)	0 to ≤ 0.10	> 0.10 to ≤ 0.50	> 0.50 to ≤ 1.00	> 1.00
Total ammonia (mg/L)	≤ 0.10	> 0.10 to ≤ 0.45	> 0.45 to ≤ 1.60	> 1.60
Escherichia coli *(E. coli*) (CFU/100 mL)	≤ 100	> 100 to ≤ 300	> 300 to ≤ 500	> 500
Enterococci (CFU/100 mL)	≤ 40	> 40 to ≤ 120	> 120 to ≤ 200	> 200
Rs	100	75	50	0

#### Weighting of Parameters

The relative weights of the selected water quality parameters were determined using the Analytic Hierarchy Process (AHP)^34–36^. Pairwise comparisons were conducted using Saaty’s 1–9 scale of relative importance, where 1 indicates equal importance, and 9 indicates extreme importance of one parameter over another. The comparisons were based on each parameter’s relevance to recreational water quality, public health risk, pollution indication, and ecological significance. Microbial indicators, particularly *E. coli* and Enterococci, were given the highest priority because they are directly associated with fecal contamination and recreational health risks. Total ammonia was assigned high importance as an indicator of sewage and organic pollution, whereas nutrients, dissolved oxygen, turbidity, and pH were assigned moderate to low importance due to their indirect influence on recreational water suitability. The pairwise comparison matrix was normalized to obtain the final parameter weights, and the consistency ratio was calculated to assess the reliability of the judgments (Table 2).

**Table 2:** Weighting of the parameters for RWQI calculation.

Parameter	weight (Wi)
pH	0.025
Dissolved oxygen (DO)	0.04
Turbidity (NTU)	0.04
Total phosphorus (TP mg/L)	0.066
Total nitrogen (TN mg/L)	0.066
Total ammonia (mg/L)	0.147
Escherichia coli (*E. coli*)	0.308
Enterococci (CFU/100 mL)	0.308
Σ	1

#### Index score

Once the normalized AHP weights were obtained and rating scores assigned to each parameter, the weighted recreational water quality index was initially computed using the following formula:

$$\:{RWQI}_{Wighted}=\:{\sum\:}_{i=1}^{n}(Wi\times\:R{s}_{i})$$

where $\:{RWQI}_{Wighted}$ is the weighted recreational water quality index, *\:Wi* is the normalized weight of parameter (i), $\:R{s}_{i}$ is the rating score assigned to parameter (i), and (n) is the total number of parameters included in the index. However, because microbial indicators represent direct public health risks in recreational waters, a non-compensatory risk-based adjustment was applied. This prevents severe microbial exceedances from being hidden by acceptable physicochemical conditions. This adjustment is critical for protecting public health, as these microbial indicators are strongly associated with waterborne illnesses and pose significant health risks to recreational water users^37,38^.

Therefore, the final recreational water quality index was calculated as:

$$\:{RWQI}_{final}=max\:\left({RWQI}_{Wighted}{\mathrm{,}Rs}_{E\bullet\:..,,.\:.Coli}\mathrm{,\:}{Rs}_{Enterococci}\right)$$

Where $\:{RWQI}_{final}$ represents the final risk-based recreational water quality index, $\:{\mathrm{,}Rs}_{E\bullet\:..,,.\:.Coli}$ is the rating score for Escherichia coli, and the $\:{Rs}_{Enterococci}$ is the rating score for Enterococci. This approach ensures that the final classification reflects the highest level of health-related risk, particularly when microbial contamination exceeds recreational water quality limits.

#### Classification of the RWQI score

The RWQI classifies water quality into four categories on the basis of the index value ranges:

90–100: Excellent—Optimal conditions for recreational activities.

65–89: Good—Generally safe for recreation but may require occasional monitoring.

26–64: Poor—Not ideal for recreational use; may pose health risks.

0–25: Unsuitable for Recreational Purposes—High risk to public health; not safe for recreational activities.

### Water quality assessment

The water quality data collected from the three coastal sites—Obhur Al-Shamaliya, Obhur Al-Janoubiyah, and Al-Saif Beach (Table SI 1)—were compared against established standards from both international guidelines, such as those provided by the WHO, and local standards set by the SAAWQS^37,39^(Table 3). This comparison is conducted to evaluate the suitability of these waters for recreational use, identify any potential health risks associated with waterborne contaminants, and understand the broader implications for environmental management and public health.

**Table 3:** Water quality standard values according to the Saudi Arabia Ambient Water Quality Standards and World Health Organization.

Parameter	Unit	SAAWQS	WHO
pH	PH units	6.5–8.5	4–11
Dissolved oxygen (DO)	mg/L	5	-
Turbidity	NTU	3	-
Total phosphorus	mg/L	-	0.02
Total nitrogen	mg/L	-	0.5–1.0
Total ammonia	mg/L	0.1	-
Escherichia coli (*E. coli*)	CFU/100 ml	500	-
Enterococci	CFU/100 ml	200	200

#### pH Levels

The pH levels across all three sites ranged between 6.93 and 8.34, which is within the acceptable range of 6.5 to 8.5, as specified by both the WHO and Saudi Arabia standards (Table 3). The stability of the pH at these sites suggests no significant acidification or alkalinization, which is a positive indicator of the overall chemical stability of the water. Similar pH values have been reported by others along the Jeddah coast^8,40,41^.

#### Dissolved Oxygen (DO)

The data showed seasonal variation (Table SI 1), with some summer readings at L1 and L2 falling below this threshold, particularly on S2, where the DO concentration decreased to 3.53 mg/L at L1. Low DO levels can lead to hypoxic conditions, which are stressful for most marine organisms and can result in fish death and loss of biodiversity. The higher DO levels recorded during the winter months (lowest (6.32) and highest (7.53) mg/L at L1 and L2, respectively) indicate better oxygenation of the water, which is typically associated with lower temperatures and reduced biological activity. Other studies reported similar results for the southern part of the Jeddah coastline, which represents L3 in this study^40^. These findings underscore the need for targeted management strategies during warmer months, such as reducing organic loading and controlling nutrient inputs, to prevent oxygen depletion and protect marine life.

#### Turbidity

Turbidity levels at L2 and L1 exceeded the threshold during the summer, peaking at 8.25 and 6.05 NTU, respectively, on S2 and 7.00 at L1 on S3. The elevated turbidity levels during the summer at L2 suggest increased sediment runoff and possibly heightened recreational activities, both of which can disturb sediments. However, the turbidity at L3 was within the limit of Saudi ambient water quality standards. Al-Mur (2025) reported less turbidity at all studied locations.

#### Nutrient levels (total phosphorus and total nitrogen)

The data revealed that nutrient levels were generally higher during the summer months, reflecting increased runoff from urban areas, agricultural activities, and possibly industrial discharge. For instance, total nitrogen levels at L3 peaked at 2.67 mg/L on S3, exceeding the recommended limits and raising concerns about the potential for eutrophication. These findings emphasize the need for effective nutrient management strategies, including reducing nutrient inputs from both point sources (such as wastewater discharge) and nonpoint sources (such as agricultural runoff), to prevent the degradation of water quality and the loss of ecosystem services provided by these coastal environments.

#### Total ammonia

Most of the recorded ammonia levels across the sites were within this limit, and there were occasional exceedances, particularly at L2 during the winter months, when the ammonia concentration reached 1.025 mg/L on W1. This spike could be due to reduced water circulation in the bay, leading to the accumulation of ammonia from decomposing organic matter. These findings suggest that ongoing monitoring and management are necessary to prevent the buildup of ammonia to toxic levels, particularly in semienclosed water bodies, such as L2.

#### Microbiological indicators

The data from L2 revealed alarming levels of contamination, with *E. coli* levels peaking at 3875 CFU/100 ml on S2, far exceeding the recommended limits. However, enterococci levels did not exceed safe limits, with the highest recorded level being 40.5 CFU/100 ml on W1 at L2. The exceedances of *E. coli* indicate significant fecal contamination, likely due to urban runoff, sewage leaks, or increased human activity during the peak recreational season. The presence of these pathogens poses a serious risk to public health, particularly for swimmers and other recreational water users, and underscores the need for immediate intervention to address sources of contamination and improve wastewater management practices in the area^42,43^.

### Application of the RWQI

The RWQI was utilized to assess the water quality of three significant recreational sites along the Jeddah coastline: Obhur Al-Shamaliya Public Swimming Beach (L1), Obhur Al-Janoubiyah Public Swimming Beach (L2), and Al-Saif Beach (L3). The results of the RWQI score and water quality classification are shown in Table 4.

**Table 4:** RWQI_*fnal*_ scores and water quality classifications for the selected sites.

Sampling time	L1		L2		L3	
	Score	Quality	Score	Quality	Score	Quality
S1	83	Good	83	Good	83	Good
S2	0	Unsuitable	0	Unsuitable	0	Unsuitable
S3	86	Good	87	Good	83	Good
W1	90	Excellent	77	Good	92	Excellent
W2	98	Excellent	84	Good	93	Excellent
W3	90	Excellent	86	Good	88	Good

On S2, the water quality at L1, L2, and L3 was classified as unsuitable for recreational use because the *E. coli* concentration exceeded 500 CFU/100 mL, with recorded values of 2300, 3875, and 2,58.13 CFU/100 mL, respectively. This severe contamination event triggered the automatic classification as unsuitable, as stipulated in the RWQI methodology, regardless of other water quality parameters. The extremely high *E. coli* levels significantly reduced the subindex score for this parameter, leading to a lower overall RWQI score for that date.

In contrast, on other dates, such as W2, the RWQI score reflected an ‘Excellent’ water quality classification for L1 and L3, with high sub-index values across most parameters and no exceedance of critical microbial thresholds. The variability in water quality, particularly the summer spike in microbial contamination, aligns with findings from previous studies that highlight the susceptibility of coastal waters to pollution from increased anthropogenic activity and inadequate wastewater treatment during peak seasons.

The RWQI results for L1 and L3 indicate a consistently good to excellent water quality classification across all the sampling dates, excluding S2. The stable subindex values for most parameters, particularly for microbial indicators, such as *E. coli* and enterococci, suggest that the beach is generally safe for recreational activities. The stable water quality at this site can be attributed to effective pollution control measures and the relative isolation of the beach from significant urban and industrial runoff sources.

These findings highlight the value of the RWQI in providing a clear and standardized assessment of water quality, which can be used by environmental managers and policy-makers to make informed decisions about recreational water use and to implement targeted interventions where necessary. Moreover, the ability of the RWQI to condense complex water quality data into a single, comprehensible score makes it a powerful tool for communicating water quality issues to the public and for promoting awareness about the importance of maintaining clean and safe recreational water^44,45^.

### Seasonal variation in the RWQI and statistical significance

Table 5 presents the average RWQI_*weighted*_ values for each sampling location (L1, L2, and L3) across both summer (S1–S3) and winter (W1–W3) sampling campaigns. The table includes calculated seasonal averages, total averages, standard deviations and ranges to highlight variability and robustness in the data. Notably, L3 resulted in the highest overall RWQI_*weighted*_ (87.09), whereas L2 resulted in greater variability in summer scores (StdDev = 17.40; Range = 31.80), reflecting localized pollution spikes.

To statistically assess seasonal differences, a one-way ANOVA test was conducted. The results (F = 8.29, *p* = 0.045) indicated a significant difference in the mean RWQI_*weighted*_ scores between summer and winter (*p* < 0.05). These findings suggest that seasonal factors, including temperature, stormwater runoff, or seasonal tourist activity, significantly influence water quality. These findings support the need for season-specific water quality management strategies in recreational coastal areas.

**Table 5:** RWQI_*weighted*_ averages with seasonal variability and robustness measures.

Location	S1	S2	S3	W1	W2	W3	Summer Avg	Winter Avg	Total Avg	Summer StdDev	Winter StdDev	Summer Range	Winter Range
L1	83.13	56.30	86.08	90.10	98.35	90.38	75.17	92.94	84.24	16.41	4.69	29.78	8.25
L2	83.13	55.00	86.80	76.70	83.68	86.43	74.98	82.27	77.72	17.40	5.01	31.80	9.73
L3	83.13	79.10	83.13	91.75	93.40	88.08	81.78	91.08	87.09	2.32	2.73	4.03	5.33

The ANOVA results shown in Table 6 further confirm the statistical significance of the observed seasonal variation in the RWQI_*weighted*_. The test yielded an F statistic of 8.29, with a p value of 0.045, indicating a statistically significant difference between the RWQI_*weighted*_ values recorded in summer and winter. These results support the assertion that external seasonal factors may play a critical role in influencing microbial water quality at recreational sites.

**Table 6:** One-way ANOVA results for RWQI_*weighted*_ seasonal comparison.

Statistic	Value
F statistic	8.29
p value	0.045
Conclusion	Significant difference (*p* < 0.05)

### Potential pollution sources

Identifying the sources of water quality along the Jeddah coastline is essential for understanding the underlying causes of water degradation and developing effective management strategies. Analysis of water quality data, combined with historical records and field observations, reveals several key pollution sources: urban runoff, industrial discharge, untreated or partially treated sewage, recreational activities, and natural influences.

#### Sewage and wastewater

High levels of microbial contamination, indicated by elevated concentrations of *E. coli* and enterococci, indicate the discharge of untreated or partially treated sewage into coastal waters. This issue is particularly evident at L2 and, to a lesser extent, at L1. Jeddah’s sewage infrastructure, although extensive, is often strained during peak periods, leading to overflows or leaks that release contaminants into the environment. Additionally, illegal discharges from boats, coastal establishments, and smaller residential areas without proper sewage connections exacerbate the problem. The presence of fecal indicators, such as *E. coli*, highlights the potential health risks associated with recreational activities in these waters, as these bacteria are commonly associated with gastrointestinal illnesses and other infections^28^.

#### Recreational activities

Recreational activities, particularly during the summer months, significantly impact water quality. Swimming, boating, and jet skiing disturb sediments at the bottom of the water body and introduce pollutants directly into the water. For example, motorized watercraft can release oil and fuel residues, whereas the physical disturbance of sediments can resuspend previously settled contaminants, increasing their availability in the water column. The increased turbidity observed during peak recreational periods suggests that these activities contribute to the deterioration of water clarity and quality. Studies have shown that recreational activities can exacerbate the input of pollutants, thereby impacting overall water quality and posing risks to marine life and human health^46^.

#### Urban runoff

Urban runoff is a primary contributor to pollution in Jeddah’s coastal waters, exacerbated by rapid urbanization and the expansion of impermeable surfaces such as roads and pavements. During rainfall, these surfaces prevent water from naturally infiltrating the ground, leading to the accumulation of surface water that contains various pollutants, including oils, heavy metals, nutrients, and debris. These pollutants are then washed directly into coastal waters, particularly in densely populated areas, such as L2. The semienclosed nature of this bay further aggravates the situation by limiting water circulation, causing pollutants to accumulate rather than disperse or dilute. Research has indicated that this has significant implications for aquatic ecosystems and the health of recreational water users, as reflected by the elevated levels nutrients in coastal waters^26,29,30,47^.

#### Natural influences

While anthropogenic factors play a significant role in water pollution, natural influences such as tides, currents, and seasonal variations also contribute to the distribution and concentration of pollutants. The Jeddah coastline, particularly in the vicinity of L2, experiences relatively low water circulation because of its semienclosed geography. This characteristic means that pollutants introduced into the water are less likely to be flushed out by tidal movements, leading to higher concentrations over time. Seasonal variations, such as high temperatures during summer, further exacerbate these effects by promoting the growth of harmful microorganisms and increasing evaporation rates, which can concentrate pollutants.

### Implications for public health and environmental management

The findings of this study have substantial implications for public health and environmental management along the Jeddah coastline. Elevated levels of microbial contaminants, such as *E. coli* and enterococci, coupled with the presence of pollutants, such as heavy metals and nutrients, underscore the urgent need for targeted interventions to safeguard both human health and the marine environment.

The detection of significant amounts of *E. coli* and enterococci, particularly in the vicinity of L2, highlights the potential public health risks associated with recreational water use. These microbial contaminants are primary indicators of fecal pollution, which is strongly linked to gastrointestinal illnesses, respiratory infections, and skin diseases in humans. The observed contamination levels suggest possible failures in wastewater treatment infrastructure or illegal discharge, presenting immediate concern for the health of beachgoers, especially during peak tourist seasons^37^.

The results emphasize the need for robust environmental management practices to mitigate the identified pollution sources. The challenges posed by urban runoff, industrial discharge, and untreated sewage require a comprehensive approach to prevent further degradation of coastal waters.

Strengthening environmental regulations and ensuring strict compliance by industrial and municipal entities are crucial for improving water quality. Effective enforcement mechanisms, combined with regular monitoring, are essential to prevent illegal discharge and ensure that wastewater treatment plants operate efficiently.

These findings suggest the need to upgrade existing sewage treatment facilities to better handle increasing urban loads. Implementing advanced treatment technologies capable of removing microbial contaminants and nutrients is critical for protecting marine environments. Additionally, detecting and curtailing illegal sewage discharge from boats and coastal establishments should be prioritized.

Educating the public about the importance of water quality and the health risks associated with polluted water is essential. Public awareness campaigns can encourage responsible behaviors, such as proper waste disposal and reduced fertilizer use, thereby decreasing the pollutant load entering coastal water.

Establishing a continuous water quality monitoring program is critical for the early detection of pollution events and the assessment of long-term trends. This program should regularly sample and analyze key water quality indicators, with results made publicly available to foster community engagement and inform management decisions. Further research is necessary to identify the sources and pathways of pollution more precisely, enabling the development of targeted interventions^48^.

## Conclusion

In this study, a RWQI was developed and applied to assess the water quality of three key recreational sites along Jeddah’s Red Sea coastline: Obhur Al-Shamaliya, Obhur Al-Janoubiyah, and Al-Saif Beach. The findings provide a detailed understanding of the physical, chemical, and microbiological conditions of these coastal areas, offering insights into their suitability for recreational use and identifying key environmental challenges. The water quality at Obhur Al-Shamaliya was relatively high. Physical parameters, such as pH and dissolved oxygen, remained stable and within the acceptable limits set by international guidelines. While minimal for most of the study period, microbiological contamination showed occasional spikes in *E. coli* levels. However, these incidences were not persistent, and the site generally maintained good water quality, supporting its continued suitability for recreational purposes. In contrast, the water quality of Obhur Al-Janoubiyah was more variable. The semienclosed nature of the site, coupled with high-density residential and recreational activities, contributed to elevated nutrient levels, specifically TN, TP, and *E. coli* concentrations, which frequently exceeded safe limits. As a result, Obhur Al-Janoubiyah experienced periods of poor water quality, posing intermittent risks to recreational users. Despite its proximity to industrial zones, Al-Saif Beach generally maintained an acceptable water quality throughout the study period. However, there were occasional increases in the TN and TP levels. Overall, Al-Saif Beach demonstrated good water quality, with the RWQI indicating that it remains a safe and suitable location for recreational activities, provided that regular monitoring continues to manage occasional fluctuations. The RWQI serves as a useful policy tool because it simplifies complex data and is presented in a format that is easy for decision-makers, regulators, and the public to understand. This contributes to improving communication about health risks, supports evidence-based management, and provides a practical framework for setting priorities and implementing measures such as issuing warnings, closing beaches, or improving infrastructure. The observed seasonal variability also underscores the need for adaptive monitoring programs that consider temporal changes in recreational water quality. However, because of the limited study period, the observed seasonal patterns should be regarded as preliminary and interpreted with caution. Furthermore, extended, spatially comprehensive monitoring is necessary to confirm these results and enhance their relevance for management. Finally, the results highlight the urgent need to improve sewage infrastructure to reduce pollution risk and protect public health.

## Electronic Supplementary Material

Below is the link to the electronic supplementary material.

Supplementary Material 1

Supplementary Material 2

## Data Availability

All the data generated or analyzed during this study are included in this published article and its supplementary information files.
