# Supplementary material for: Development and evaluation of a recreational water quality index for the Red Sea Coastline, Saudi Arabia
Source: Sci Rep. 2026 May 24;16:23752. doi: 10.1038/s41598-026-54623-9 (PMC13429577; doi:10.1038/s41598-026-54623-9)

Development and Evaluation of Recreational Water Quality Index for Red Sea Coastline, Saudi Arabia

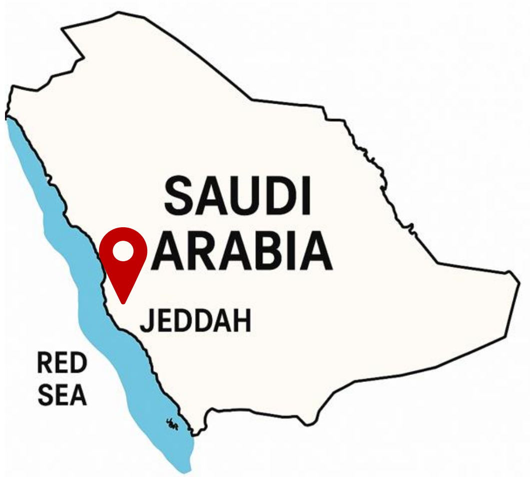

Parameters Selection

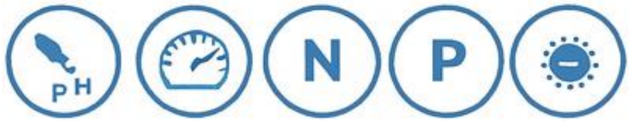

pH DO TN TP *E coli*

Weighting of Paramerts

Micorbial Indicators = 3

Nutrients =2

Physicochemical parameters =1

RWQI calculation

$$WQI = \sum_{i=1}^{n} (Wi \times Rs_i)$$

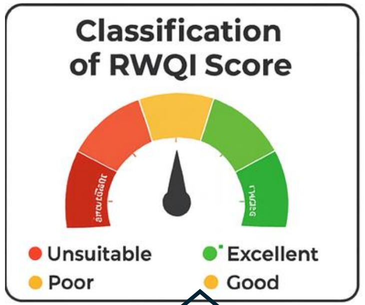

Rating Scale

Excelient	Good	Foor	Unsuitable
90-100	65-89	26–64	0–25

→ If *E.* coli >500 CFU/100 mL or Enterococci >200 CFU/100 mL → Automatically Unsitiab

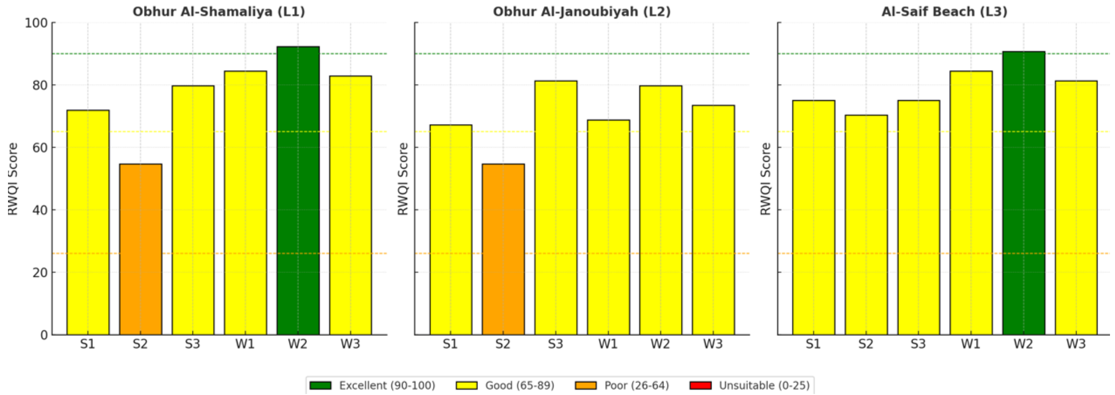

Supplement: Supplementary file 2 — Supplementary Material 2 [file 41598_2026_54623_MOESM2_ESM.pdf]
